# Cobalt-doped copper vanadate: a dual active electrocatalyst propelling efficient H_2_ evolution and glycerol oxidation in alkaline water†

**DOI:** 10.1039/d2na00724j

**Published:** 2022-11-25

**Authors:** Vijay Tripathi, Siddarth Jain, Dinesh Kabra, Leela S. Panchakarla, Arnab Dutta

**Affiliations:** Department of Chemistry, Indian Institute of Technology Bombay Mumbai 400076 India arnab.dutta@iitb.ac.in jainsiddarth477@gmail.com; Department of Physics, Indian Institute of Technology Bombay Mumbai 400076 India; Interdisciplinary Program in Climate Studies, Indian Institute of Technology Bombay Mumbai 400076 India; National Center of Excellence in CCU, Indian Institute of Technology Bombay Mumbai 400076 India

## Abstract

Strategically doped metal oxide nanomaterials signify a rapidly growing genre of functional materials with a wide range of practical applications. Copper vanadate (CuV) represents one such highly active system, which has been rarely explored following its doping with an abundant first-row transition metal. Here, we have developed a series of CuV samples with varying cobalt(ii) doping concentrations deploying a relatively simple solid state synthetic procedure. Among the samples, the 10% Co(ii)-doped CuV (Co_10%_–CuV) exhibited excellent reactivity for both the H_2_ evolution reaction (HER) and glycerol oxidation reaction (GOR) in an alkaline aqueous medium (pH 14.0) during cathodic and anodic scans, respectively. During this dual-active catalysis, surface-immobilized Co_10%_–CuV operates at exceptionally low overpotentials of 176 mV and 160 mV for the HER and GOR, respectively, while achieving 10 mA cm^2^ current density. The detailed spectroscopic analysis revealed the formation of formate as the major product during the GOR with a faradaic efficiency of >90%. Therefore, this Co_10%_–CuV can be included on either side of a two-electrode electrolyzer assembly to trigger a complete biomass-driven H_2_ production, establishing an ideal carbon-neutral energy harvest process.

## Abstract 2

Co doping resulted in an enhancement of the electrocatalytic properties of copper vanadate towards the HER and the GOR to form formic acid.

## Graphical abstract

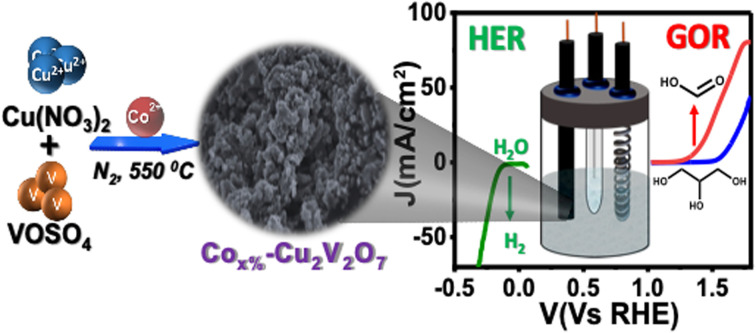

## Introduction

Transition metal oxides represent a unique genre of multi-functional materials driving a variety of advanced practical applications, including optoelectronics, energy storage, sensors, and photocatalysts.^1–3^ Further modulation of the morphology of these metal oxides further widened the scope of their operations as newer avenues of syntheses and tailored processes originated. The addition of dopants has recently emerged as a new synthetic tool to finetune metal oxide lattices by altering the interstitial defects or oxygen vacancies. Such modifications cause phase transitions at higher concentrations or even structural deformations in unstable host lattices, resulting in a shift in their physical and chemical properties. Rational doping of Co(ii), Zn(ii), and Ni(ii) ions into metal oxides, perovskites, and tungstate and vanadate frameworks is a primary example of this approach as this offers improved magnetic and photovoltaic properties.^4–6^ Similarly, optimum lanthanide doping in BaWO_4_ for developing a glucose detection sensor and zinc or antimony doping in TiO_2_ for improvised sensing and photonic applications are also worth mentioning.^7,8^ Hence, selecting dopants and robust template materials such as Fe_2_O_3_, ZnO, Na_2_WO_4_, and Na_2_MoO_4_ is key to originating new materials.^8–10^ Ideally, the best results are observed when the dopant concentration remains on the lower side and does not induce any significant changes in the base material lattice structure, leading to a series of modular designs for a compliant range of applications.^11–14^

Metal oxides can be constituted of multiple components present in either stoichiometric or non-stoichiometric amounts, harbouring several cations in divergent coordination geometries. Therefore, metal oxide templates can support the facile transformation of different metal oxidation states, which is vital for creating reaction hot spots on the surface of the material leading to unique electrocatalytic capabilities. Metal vanadate is an exciting class of metal oxides that can generate numerous reactive states to be useful for direct applications in the fields of electrocatalysis, photocatalysis, energy storage systems, magnetic devices, *etc.*^15–19^ Copper vanadates are one of the top choices in this regard, which can exist in variable conformations, such as CuV_2_O_6_, Cu_2_V_2_O_7_, Cu_3_V_2_O_8_, Cu_11_V_6_O_26_, and Cu_5_V_2_O_10_ while displaying a range of p and n-type semiconductor phases.^20–24^ Copper vanadates have gained attention due to their structural versatility (nanostructure or film) and tunable electrochemical and photocatalytic properties. The strategic alterations of reaction temperature, synthesis method, reactants, and other reaction conditions minutely tune the different phases of these particular vanadates. The critically developed CuV_2_O_6_ nanoparticles and nanobelts have found their usage as electrode materials in batteries.^21,22^ On the other hand, Cu_3_V_2_O_8_ was employed for photocatalysis and water splitting.^23^ Cu_5_V_2_O_10_ contains distorted Cu(ii) ions, which were instrumental in showcasing unique magnetic anisotropy.^24^ Nickel vanadate (Ni_3_V_2_O_8_) was doped with Co(ii) to craft CoNi_2_V_2_O_8_ that can be potentially implemented as an electrode material for batteries and supercapacitors;^25^ whereas tungsten or molybdenum doping in bismuth vanadate leads to its improved and efficient performance as a photo-electrode and in electrochemical applications.^26^ Cu_2_V_2_O_7_ has also been substantially investigated as a photo-anode material, while its transition metal doped counterparts such as Co–Mg doped and Zn doped Cu_2_V_2_O_7_ have demonstrated multiferroic and dielectric properties.^27,28^ Cu_2_V_2_O_7_ was also doped with the divalent 3d-transition metals Zn(ii) and Mn(ii) to further explore their prospective applications in photocatalysis. Despite the commendable progress in this realm, the specific influence of doped copper vanadates is mostly restricted to photoanode, supercapacitor, battery, and photocatalyst-based applications.^29–33^ The potential of these otherwise unusual materials has been rarely probed for electrochemical reductions (cathodic) and organic molecule oxidations.

The current energy scenario and the negative impacts of excessive fossil fuel usage have exacerbated environmental pollution challenges and compelled human society to explore alternative solutions leading to sustainable development. The utilization of renewable energy and biomass has been reckoned to be one of the best options in pursuing a carbon-neutral energy landscape. The storage of intermittent renewables into a chemical vector H_2_ is a key step, where the presence of a hydrogen evolution reaction (HER) catalyst is essential. On the other hand, a catalyst capable of oxidizing the reduced carbon sources and extracting the stored energy is vital for harnessing biomass energy. However, copper vanadates have rarely been probed as electrocatalysts for the electrocatalytic HER and organic molecule oxidation reactions. However, copper vanadate provides a robust and conductive template, which can be activated with strategic doping with appropriate materials. In this regard, cobalt ions offer an excellent option. The widely abundant and inexpensive cobalt ions are well-known for their role in supporting electrocatalytic water-splitting reactions. Recent research reveals that the electrocatalytic efficacy of the HER is enhanced by the presence of cobalt ions owing to the favourable hydrogen adsorption energy of their interfacial cobalt sites (compared to those of other 3d metals such as Ni and Fe). Additionally, cobalt ions also promote water adsorption and dissociation on the catalyst surface to drive the water oxidation reaction.^34,35^ Similarly, the role of cobalt-based catalysts in promoting glycerol oxidation into various value-added products (C1–C3) has been explored lately.^36,37^

**Scheme 1:**
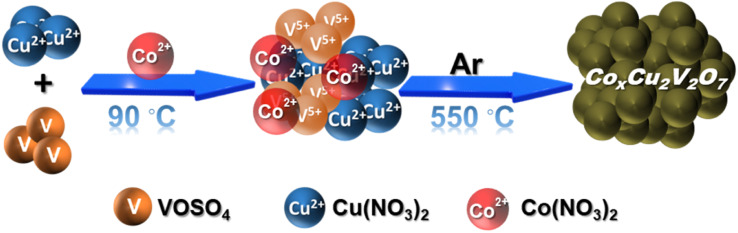
Schematic illustration showing the formation method of Co^2+^ doped Cu_2_V_2_O_7_.

Here, in this work, we have rationally incorporated a Co(ii) dopant in a copper vanadate (Cu_2_V_2_O_7_) framework to explore its capability of driving both the HER and glycerol (a reduced carbon resource) oxidation (Scheme 1). The 10% cobalt doped electrocatalyst was screened to be the optimized electrocatalyst that demonstrated excellent HER (overpotential 176 mV @ 10 mA cm^−2^) as well as glycerol oxidation (overpotential 160 mV @ 10 mA cm^−2^) in an alkaline aqueous solution (pH = 14). During the oxidation reaction, formic acid was found to be the major product, which is reckoned as a crucial C_1_ feedstock in the chemical industry. Therefore, developing this new class of dual-active electrocatalysts can establish a solid foundation for designing devices capable of simultaneously generating fuel and processing biomass.

## Results and discussion

**Fig. 1:**
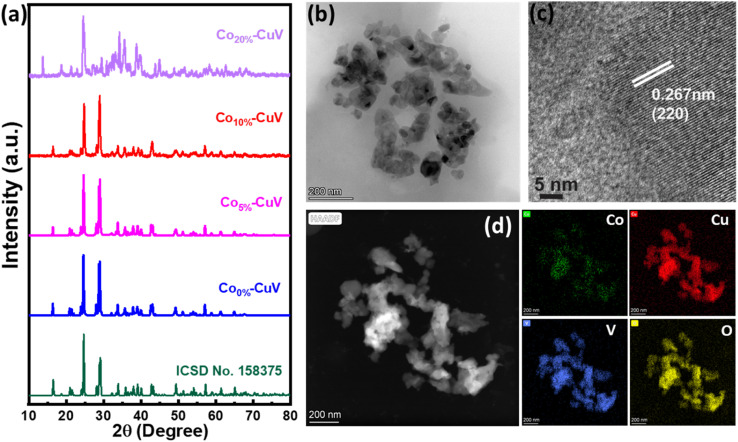
(a) Powder XRD patterns of CuV and Co_*x*%_–CuV; (b) TEM image of Co_10%_–CuV; (c) HR-TEM image of Co_10%_–CuV; (d) HAADF image and elemental mapping of Co_10%_–CuV obtained at 550 °C under an inert atmosphere.

A series of Co(ii) doped copper vanadate was prepared by adding appropriate amounts of Cu(NO_3_)_2_ into a mixture of VOSO_4_ and Co(NO_3_)_2_ during the solid-state synthesis executed at 550 °C for two hours under an inert atmosphere. The pristine copper vanadate sample was also prepared in the same process that produced β-Cu_2_V_2_O_7_ species (Co_0%_–CuV). The cobalt content varied in the doped samples at 5, 10, and 20 mol percent, which were denoted as Co_5%_–CuV, Co_10%_–CuV, and Co_20%_–CuV, respectively. Here, the cobalt doping was stopped at 20% as a further increase in Co(ii) composition leads to a permanent phase change of copper vanadate. Furthermore, the role of inert reaction conditions in the sample preparation was also investigated. To explore this feature, another series of reactions were set up, only varying the atmosphere (from anerobic to aerobic) while keeping the rest of the conditions identical. The effect of aerial oxygen was evident from the generation of a major CuO phase along with copper vanadate (Fig. S1†). Initially, the systematic structural analysis of these samples was performed to gauge their morphological divergence. Fig. 1a depicts the XRD patterns of synthesized pure copper vanadate (Co_0%_–CuV) and Co(ii) doped CuV samples (Co_0–20%_–CuV). The obtained XRD spectra of all the samples supported the presence of a crystalline monoclinic β-Cu_2_V_2_O_7_ structure (ICSD no. 158375), except for Co_20%_–CuV. The relatively higher Co(ii) doping proportions (>10%) exhibited additional signals in the XRD spectra, which could not be indexed to the β-Cu_2_V_2_O_7_ phase of copper vanadate. These new peaks possibly indicated the formation of new phases following Co(ii) doping. The sharpness and intensity of these XRD peaks suggested a high degree of crystallinity in the samples. No XRD signature peaks were observed during this study that can be attributed to metallic cobalt or cobalt oxides. This result demonstrated the purity of the as-prepared samples, indicating that Co(ii) was successfully incorporated into the crystalline monoclinic copper vanadate (Co_0%_–CuV) lattice sites. Interestingly, the XRD peak intensities and corresponding line widths altered slightly after Co(ii) ions were inserted in the CuV host lattice.

The structural configuration of pure CuV and Co(ii)-doped CuV (Co_0–20%_–CuV) were investigated further using SEM (Fig. S2a–d†). The morphology of pure copper vanadates (Co_0%_–CuV) revealed the existence of agglomerated nanoparticles in clusters with an average diameter of 30–40 nm, indicating a homogeneous growth of nanoparticles (Fig. S2a†). SEM images obtained from Co(ii) samples displayed no significant change in particle size or morphology, showcasing the conservation of the same copper vanadate framework (Fig. S2b–d†).

Next, the morphology of the model Co(ii)-doped sample (Co_10%_–CuV) was studied by TEM analysis, which reaffirmed the formation of 30–40 nm diameter nanoparticle formation (Fig. 1b). HR-TEM was employed to gain a better insight into the Co_10%_–CuV structure. The lattice fringes of Co_10%_–CuV revealed a highly crystalline phase with a *d*-spacing of 0.26 nm, inferring growth along the (220) plane (Fig. 1c). In addition, there are no secondary structures or impurities evident in the image, indicating that all Co(ii) ions are uniformly integrated into the copper vanadate template without any cobalt clustering. The TEM-EDS data confirmed the uniform distribution of Co, Cu, V, and O elements in the copper vanadate matrix (Fig. 1d and S3†). The dopant concentration in the copper vanadate matrix was precisely measured using inductively coupled plasma-atomic emission spectroscopy (ICP-AES), where the Co_10%_–CuV sample registered 9.6% doping of cobalt ions.

**Fig. 2:**
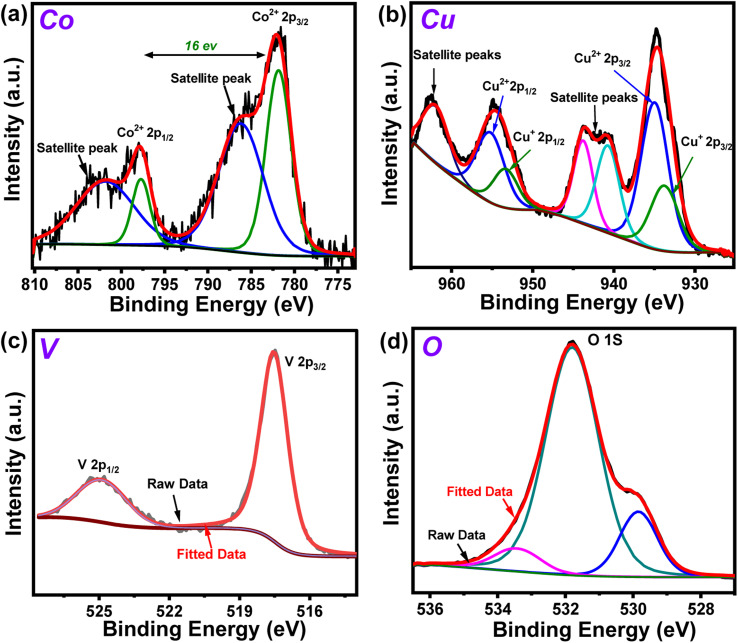
High resolution XPS spectra of (a) Co 2p, (b) Cu 2p (c) V 2p and (d) O 1s regions of the 10% Co(ii)-doped Cu_2_V_2_O_7_ (Co_10%_–CuV) sample prepared at 550 °C under an inert atmosphere.

The X-ray photoelectron spectroscopy (XPS) experiment was performed on the Co_10%_–CuV sample next to probe the chemical environment of each element. The full scan XPS spectrum reiterated the presence of all the precursor elements in the matrix with the signature Co 2p, Cu 2p, V 2p, and O 1s peaks (Fig. S4†). The Co 2p_3/2_ and Co 2p_1/2_ signals of the sample were observed at 782 and 798 eV, respectively. The occurrence of this 16 eV difference between Co 2p_3/2_ and Co 2p_1/2_ suggests that dopant cobalt ions primarily exist in the sample in the Co(ii) oxidation state.^6,38^ These signals were also accompanied by the higher energy shakeup satellites (2p_3/2_, 785.1 eV and 2p_1/2_, 802 eV) (Fig. 2a). The oxidation state of Cu in the Co_10%_–CuV host lattice was also validated using XPS data. The high-resolution XPS spectrum of the Cu 2p region exhibits prominent peaks positioned at 934.9 (Cu 2p_3/2_) and 955.2 eV (Cu 2p_1/2_), which can be ascribed to the presence of Cu(ii) ions (Fig. 2b). The satellite peaks located at 942.9 and 962.6 eV indicate an open 3d^9^ shell in the structure.^39,40^ Furthermore, shoulder peaks at lower binding energies were also noticed for both the Cu 2p_3/2_ and Cu 2p_1/2_ signals. These peaks may be attributed to reduced Cu(i) sites, which were probably generated to compensate for the oxygen vacancies present in the copper vanadate framework.^41^ The existence of the V(v) chemical state in the matrix of Co_10%_–CuV was verified by the primary V 2p asymmetric peaks with binding energies of 524.9 (V 2p_3/2_) and 517.4 eV (V p_1/2_) (Fig. 2c).^39^ The O 1s region comprises three components: a small peak at around 529.8 eV owing to oxygen and defects and two wider peaks at 531.7 and 533.4 eV originating from lattice oxygen Cu–O and V–O respectively (Fig. 2d).^42,43^

### Electrochemical HER and glycerol oxidation to formic acid

Hydrogen is currently viewed as a sustainable alternative for the currently employed fossil fuels.^44–46^ Its high energy density, absence of combustion by-products, and carbon-based energy transport provide it with an undoubtedly edge for future energy applications. In this context, electrochemical water splitting is considered a feasible approach for pure hydrogen production. Therefore, the development of low-cost and high-performance alkaline HER catalysts is vital. On the other hand, the oxygen evolution reaction (OER) completes the overall water-splitting process. This counter OER negatively impacts the efficiency of the water to H_2_ owing to the sluggish kinetics and associated high thermodynamic barrier of O_2_ evolution catalysis. In an attempt to resolve this issue, the OER can be replaced with the oxidation of reduced organic molecules (urea, hydrazine, ammonia, glycerol, ethanol, *etc.*) in the anodic compartment. Among the possible options, glycerol has emerged as the leading one owing to its prominent abundance as a side product of the bio-diesel manufacturing process. Additionally, the oxidation of glycerol leads to the generation of formic acid, which can produce additional energy with the appropriate use of formic acid fuel cells (direct and indirect).^36,47,48^ Therefore, the electrooxidation of glycerol (glycerol oxidation reaction/GOR) to formic acid is reckoned as an interesting proposition. Therefore, the electrocatalytic activity of Co(ii)-doped copper vanadate was explored for both these renewable energy-relevant reactions: the HER and GOR.

### Hydrogen evolution reaction

**Fig. 3:**
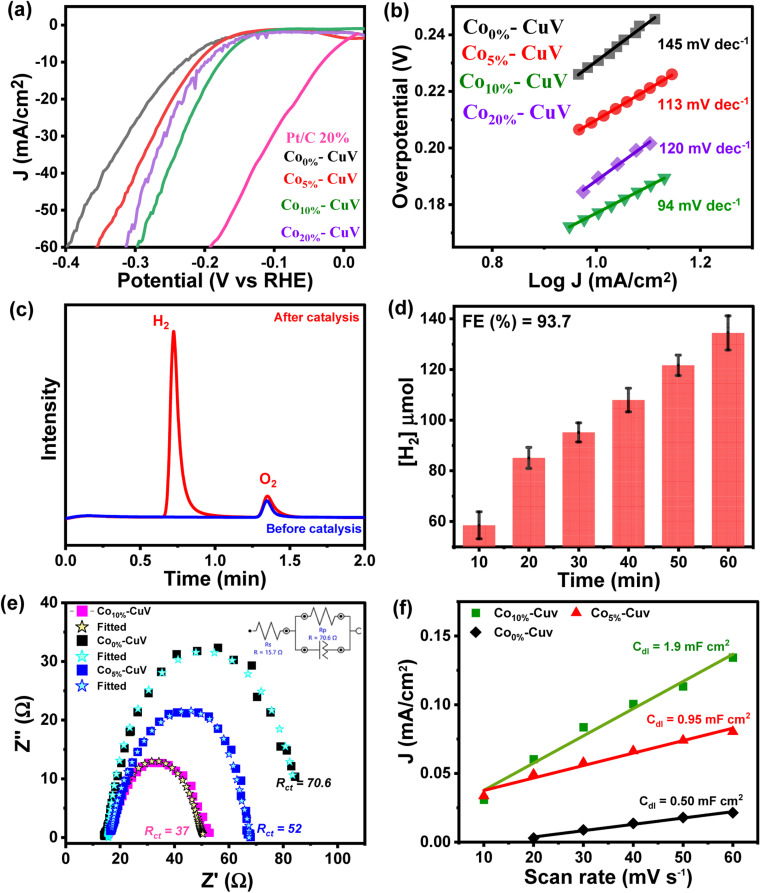
(a) Comparative linear sweep voltammograms (LSVs) and (b) Tafel plots for the HER on modified GCEs comprising pure CuV (Co_0%_–CuV) (black trace), Co-doped copper vanadates (Co_5%_–CuV) (red trace), Co_10%_–CuV (green trace), and Co_20%_–CuV (violet trace) in 1.0 M KOH. A standard three electrode set up consisting of a copper vanadate immobilized glassy carbon disc (5 mm diameter) working electrode, Pt wire counter electrode, and Ag/AgCl (3.0 M KCl) reference electrode was used here at 298 K under 1 atm N_2_. A scan rate of 5 mV s^−1^ was maintained for all the experiments. (c) Gas chromatography (GC) analysis during chronoamperometry of Co_10%_–CuV for the HER [before electrocatalysis (blue trace) and after electrocatalysis (red trace)], and (d) the serial evolution of H_2_ during the bulk electrocatalysis by Co_10%_–CuV during the bulk electrolysis experiment. (e) The comparative EIS spectra of Co(ii) doped modified electrodes were measured in 1.0 M KOH solution at an open circuit potential and with input frequencies ranging from 100 kHz to 0.01 Hz. (f) Capacitive currents for doped and undoped copper vanadate at 0.05 V as a function of scan rate.

**Fig. 4:**
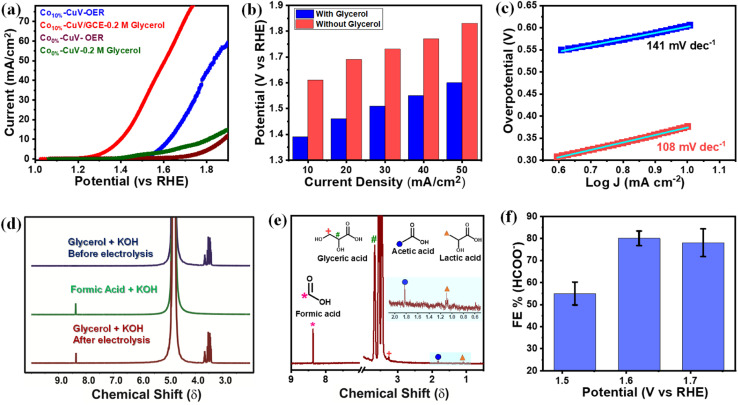
Electrochemical glycerol oxidation performance. (a) Comparative linear sweep voltammograms (LSVs) recorded at a scan rate of 5 mV s^−1^, in the presence (red trace) or absence (blue trace) of glycerol; (b) the bar plot showing input potential at different current densities for the GOR (red trace) and OER (blue trace); (c) Tafel slopes of a Co_10%_–CuV modified electrode in the presence (red dots and sea green trace) or absence of glycerol (blue dots and sky blue trace); (d) ^1^H NMR spectra of the electrolytes (0.2 M KOH + 0.2 M glycerol) and standard formic acid before and after 8 h of electrolysis at 1.6 V *vs.* RHE (D_2_O as a solvent at room temperature). The intensity of ^1^H NMR peaks for glycerol did not considerably decrease following electrolysis because of the higher starting glycerol content; (e) high resolution ^1^H NMR after 8 hours of electrocatalysis of glycerol (water peak was eliminated) (symbols showing the corresponding protons) and (f) faradaic efficiency (FE) for formic acid formation at different applied potentials (each data point was recorded following 8 hours of continuous electrolysis).

The doping effects of Co(ii) (Co_5%_–CuV, Co_10%_–CuV, and Co_20%_–CuV) on copper vanadate-derived electrocatalytic HER performance in an alkaline aqueous solution (pH 14.0, 1.0 M KOH) were evaluated by deploying a standard three-electrode system. Here, the copper vanadate derivatives were immobilized on a glassy carbon working electrode. The LSV curves (polarization curves) and Tafel slope spectra for the HER of pure CuV and Co_*x*%_–CuV are displayed in Fig. 3a and 4b. All the potential values are reported against the reversible hydrogen electrode (RHE) in this study unless otherwise mentioned. All the samples displayed a sharply reducing current response during the cathodic scan initiated at 0 V (*vs.* RHE). However, the onset potential for the HER varied significantly in the presence of the Co(ii) dopant. Pure CuV (Co_0%_–CuV) initiates H_2_ evolution at the highest cathodic potential in this series as it attained 10 mA cm^−2^ current density at an applied overpotential (*η* @ 10 mA cm^−2^) of 232 mV (*E*_H^+^/H_2__ = 0 V *vs.* RHE). The inclusion of Co(ii) improves the energy efficiency of the process which was evident from the relatively lower overpotential for the HER. The observed *η* @ 10 mA cm^−2^ values for Co_5%_–CuV, Co_10%_–CuV, and Co_20%_–CuV were 210, 176, and 199 mV, respectively (Fig. 3a). Thus, the 10% Co(ii)-doped sample (Co_10%_–CuV) emerged as the best HER catalyst. One useful metric that can shed light on the HER process is the Tafel slope.^49^ Below, we compare the two different mechanisms that could be at play.

(i) Volmer–Heyrovsky mechanism:H_2_O + e^−^ + M* ↔ MH* + OH^−^ (Volmer reaction, *b* ∼ 120 mV dec^−1^): H_2_O dissociationMH* + H_2_O + e^−^ ↔ H_2_ + M + OH^−^ (Heyrovsky reaction, *b* ∼ 40 mV dec^−1^): H desorption

(ii) Volmer–Tafel mechanism:H_2_O + e^−^ + M* ↔ MH* + OH^−^ (Volmer reaction): H_2_O dissociationMH* + MH* ↔ H_2_ + 2M (Tafel reaction *b* ∼ 30 mV dec^−1^): H recombination

The Tafel slope will be ∼120, ∼40, or ∼30 mV dec^−1^ if the rate-determining step is a Volmer, Heyrovsky, or Tafel reaction, respectively.^50^ This catalyst also showcased the most facile kinetics which was corroborated by the corresponding Tafel slope. The Tafel slopes for Co_0%_–CuV, Co_5%_–CuV, Co_10%_–CuV, and Co_20%_–CuV are found to be 145 mV dec^−1^, 113 mV dec^−1^, 94 mV dec^−1^, and 120 mV dec^−1^, respectively (Fig. 3b). Throughout this investigation, the Tafel slopes have been found to be greater than 40 mV dec^−1^, demonstrating that the HER is governed by the Volmer–Heyrovsky mechanism. Next, the bulk electrolysis (chronocoulometry) experiment was performed with the Co_10%_–CuV sample (immobilized on a carbon paper working electrode) in an air-tight cell while maintaining a constant applied potential of −0.2 V. The head-space gas was collected periodically during this experiment and analyzed *via* a gas chromatography (GC) instrument. The GC data confirmed the evolution of H_2_ during the reduction (Fig. 3c and 4d). This data also indicated an excellent faradaic efficiency (FE) of 93.7% by Co_10%_–CuV during the electrocatalytic HER. The electrocatalytic HER activity showcased by Co_10%_–CuV matched the proficiency of state-of-the-art H_2_ production catalysts in alkaline media in terms of applied overpotential (*η* @ 10 mA cm^−2^) and kinetics (Table S1†). Comparative LSV data were also collected with the same Co_10%_–CuV electrode following bulk electrolysis (at −0.2 V), which matched the pre-electrolysis HER response (Fig. S5a†). The long-term electrochemical durability of the Co_10%_–CuV modified electrode, which demonstrated a high HER performance, was further examined *via* chronoamperometry (*j vs. t* measurement). Fig. S5b† shows long-term chronoamperometry stability (up to 4 h, at a constant bias of −0.2 V) without current density decay, validating electrochemical endurance under practical conditions.

The Co(ii) dopant tends to replace the Cu(ii) from the host copper vanadate lattice. This ion replacement creates a disparity in the original skeletal structure and leads to oxygen vacancies to balance the overall charge of the lattice. These induced vacancies presumably produce active sites for enhanced electrocatalytic activity. Furthermore, to understand the significance of cobalt doping in enhancing the intrinsic electrocatalytic activity of Co_10%_–CuV for the HER, we studied the link between the Co ions and electrochemical kinetics (EIS study), as well as the active electrochemical surface area (ECSA) of the catalyst during the HER. As shown in Fig. 3e, electrochemical impedance spectroscopy (EIS) measurements were undertaken. As depicted in Fig. 3e (inset), the charge transfer resistance (*R*_ct_) and solution resistance (*R*_s_) are determined by fitting the plots using a simplified Randles circuit model. 10% Co(ii) doped CuV has a significantly lower *R*_ct_ (37) than the 5% Co(ii) doped CuV (52) pure CuV (70), indicating faster charge transfer *via* the catalytic electrode and, consequently, a rapid reaction rate in electrocatalytic kinetics. This also indicates the strong conductivity of the Co(ii) doped CuV modified electrode, which speeds up reactions and hence decreases the HER overpotential. The solution resistance (*R*_s_) has a specific value of around 14.7 ohm. The effective ECSA of the pure CuV and doped CuV samples was estimated by calculating the double-layer capacitance (*C*_dl_), which is a typical way to measure the interfacial area between the electrolyte and electrode surface, recording to the CV curves at various scan rates.^51,52^ The current density *vs.* scan rate graphs in Fig. S6† show that 10% Co(ii) doped CuV has a *C*_dl_ value of 1.9 mF cm^−2^, which is greater than that of 5% Co(ii) doped CuV (0.95 mF cm^−2^) and undoped CuV (0.5 mF cm^−2^) and suggests that 10% Co doped CuV has a higher ECSA and a greater number of active sites for the HER. The effect of cobalt doping on electrocatalytic performance was also validated by an optical band gap study (Tauc-plot) of Co_0%_–CuV (2.5 eV), Co_5%_–CuV (2.13 eV), and Co_10%_–CuV (2.05 eV) respectively (Fig. S7†). The presence of Co(ii) ions significantly lowered the band gap of the pure Co_0%_–CuV (2.5 eV), as the 5% and 10% cobalt-doped samples illustrated 2.13 and 2.05 eV band gaps, respectively. Therefore, a specific amount of Co doping drops the band gap and surface electronic structure of doped copper vanadates implying an improved electrical contact and lowered electric resistance, which enhances the charge transfer kinetics to boost the heterogeneous HER.^34,35,53^

### Glycerol oxidation reaction (GOR)

The copper vanadate derivatives were also probed for their electrooxidation during an anodic scan. The initial study was performed in an unadulterated alkaline aqueous medium (1.0 M KOH, pH 14.0), where the undoped Co_0%_–CuV sample displayed a slight change in oxidation current at around 1.4 V before showcasing a sharp increase beyond 1.7 V (Fig. 4a). The signatures are attributed to metal-based oxidation followed by catalytic water oxidation.^48,54–56^ The inclusion of cobalt (Co_10%_–CuV) shifted both the metal oxidation and onset potential for water oxidation to the cathodic direction. These changes indicate the relative ease of electron exchange during the oxidations in the presence of a dopant. A significant change was observed when 0.2 M glycerol was added to the solution. The 10% Co(ii)-doped CuV (Co_10%_–CuV) sample showcased a steep increase in the oxidation current initiating at 1.3 V before reaching 10 mA cm^−2^ current density at 1.39 V (Fig. 4a). This current response was only visible in the presence of glycerol, as evident from the high current density observed compared to the competitive OER at similar potentials (Fig. 4b). Additionally, the Tafel slope for oxidation of glycerol was determined to be 108 mV dec^−1^ for Co_10%_–CuV, which is lower than that of the OER (141 mV dec^−1^), suggesting faster kinetics for glycerol oxidation compared to the OER (Fig. 4c). In contrast, the rest of the Co(ii)-doped CuV (Co_5%_–CuV, and Co_20%_–CuV) derivatives display poorer GOR performances compared with Co_10%_–CuV (Fig. S8†). So, for this investigation, the Co_10%_–CuV sample was deemed to be the ideal sample. Pure copper vanadate also displayed the glycerol oxidation signature, albeit in a cathodically shifted region (onset potential of 1.55 V, 10 mA cm^−2^ current density and potential of 1.62 V). The improved oxidation reactivity of the Co_10%_–CuV catalyst in the presence of glycerol compared to undoped copper vanadate was also supported by a significantly lower electric impedance recorded during the electrochemical impedance spectroscopy (EIS) experiment (Fig. S9†). All these data pointed to an elevated reactivity of the Co_10%_–CuV sample for the electrocatalytic GOR.

We also evaluated the ECSA normalized activity and turn over frequency (TOF) in order to examine the intrinsic catalytic GOR activity of the cobalt-doped CuV electrocatalysts. The results are shown in Fig. S10 and S11.† The CuV with 10% Co(ii) doping has the highest values. After normalizing the GOR LSV polarization curves using their respective ECSAs (Fig. S10†), 10% Co(ii) doped hybrids show the lowest onset potential and a greater current density than the undoped copper vanadate, showing that Co_10%_–CuV has the best electrocatalytic performance per active unit for the GOR process compared to the undoped sample. Turnover frequency (TOF) may indeed be utilised to assess the intrinsic activity of electrocatalysts with more precision when their active sites are well identified. Previous experiments have clearly shown that Co(ii) ions play a crucial role in facilitating the Co(ii) doped CuV sample to carry out the GOR electrocatalysis. While identifying the active sites in single-element catalysts is relatively straightforward, multi-element composite catalysts provide unique challenges due to a lack of comprehensive techniques and understanding of catalytic mechanisms. A generally established strategy is to assume all metal sites to be active, despite the fact that this would result in an underestimation of the TOF. The TOF values (at 1.6 V *vs.* RHE) of catalysts were computed and compared as shown in Fig. S11† based on the number of active sites (postulating Co combined with Cu and V) determined by ICP-AES. Co_10%_–CuV reveals a much larger TOF in comparison to undoped copper vanadate, similarly to the ECSA-normalized current density. These findings indicated that Co_10%_–CuV has a higher innate activity toward the GOR compared to other composites.

^1^H nuclear magnetic resonance (^1^H NMR) spectroscopy was deployed to identify the glycerol oxidation products following bulk electrolysis of Co(ii)-doped copper vanadate. In this experiment, relatively modest potentials of 1.6 and 1.7 V were applied to limit the interference from the competitive OER. Formic acid is identified as the primary product of the Co_10%_–CuV-catalyzed GOR, as evident from the comparative ^1^H NMR spectra recorded before and after the electrolysis (Fig. 4d and e). The ^1^H NMR peak of formic acid was standardized further to determine the faradaic efficiency (FE) of the electrocatalytic GOR (Fig. S12†). This investigation confirmed the generation of formic acid with an FE of 79.8% at 1.6 V, which is comparable to that in previously reported work (Table S2†).^55^ The ^13^C NMR analysis was executed following the bulk electrolysis to further verify the glycerol oxidation products. The formate ions emerged as one of the major products, along with substantial amounts of carbonate (Fig. S13†). The ^1^H NMR spectra illustrated the presence of glyceric acid, lactic acid, and acetic acid, albeit at lower amounts compared to formate (Fig. 4e). These intermediate products provide us hints to decipher the glycerol oxidation mechanism, which is addressed in the subsequent segment. The applied potential has a direct effect on the product selectivity, as the FE for formic acid dropped on either side of 1.6 V (the applied potential) (Fig. 4f). The lack of available energy typically slows down the GOR at 1.5 V, while at 1.7 V, the competitive OER kicks in.

**Scheme 2:**
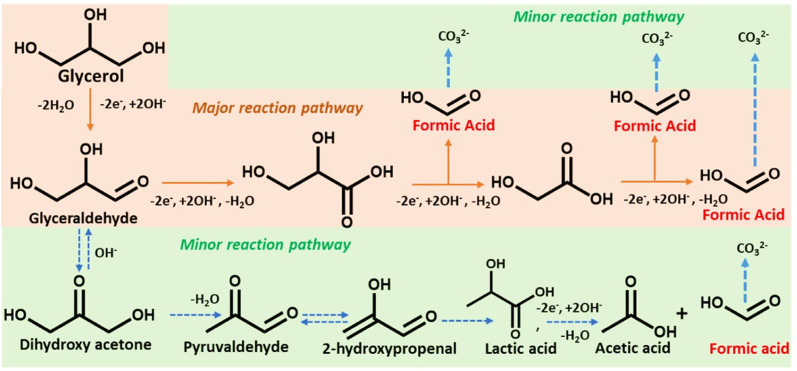
The proposed glycerol electrooxidation pathway. The orange box highlights the major reaction pathway and the green region indicates the minor reaction pathway of glycerol oxidation triggered by Co-doped copper vanadates.

Scheme 2 depicts a probable route for the electrochemical oxidation of glycerol to formate in an alkaline solution based on experimental findings and earlier reports.^48^ Electron-rich glycerol initially oxidizes to glyceraldehyde following a two-electron oxidation triggered by the Co(ii)-doped copper vanadate. The highly reactive glyceraldehyde readily converts to glycerate. The formation of glycerate during the reaction was corroborated by ^1^H NMR spectroscopy (Fig. 4e). This glycerate intermediate undergoes a C–C cleavage, followed by a two-electron transfer, resulting in the formation of equal quantities of formate and glycolate. The electron-rich glycolate continues on the oxidative track to generate another molecule of formate. The glycerol to formate oxidation can also follow an alternative pathway, where glyceraldehyde serially converts to dihydroxyacetone, pyruvaldehyde, 2-hydroxypropenal, lactic acid, and finally acetic acid. The possible presence of this minor oxidation pathway was indicated by the signature ^1^H NMR signals of acetate and lactate (Fig. 4e). Hence, Co_10%_–CuV represents one of the leading GOR electrocatalysts showcasing excellent product selectivity and energy efficiency.

Long-term stability of the catalysts under electrocatalytic conditions is a key factor for developing practical assemblies with a higher technology readiness level (TRL). For the lifetime measurement of the Co_10%_–CuV electrode, an 8 hour long continuous chronocoulometry experiment was performed at an applied potential of 1.6 V. During the experiment the current response changes up to (∼30%) over the course of the experiment (Fig. S14†). Comparative LSV data were also recorded with the same Co_10%_–CuV electrode post chronocoulometry, where the electrocatalytic GOR activity was retained by the electrode (Fig. S15†). To probe the repeatability of the bulk GOR activity, the Co_10%_–CuV modified electrode was deployed for electrocatalytic glycerol oxidation for five continuous cycles of bulk electrolysis (applied potential 1.6 V, duration 1 hour). Impressively, the Co_10%_–CuV electrode continued to oxidize glycerol with minimal loss (<5%) during this experiment (Fig. S16†). Therefore, this Co_10%_–CuV sample demonstrates the potential of direct inclusion for practical devices driving both the electrocatalytic HER and GOR.

## Conclusion

In summary, monoclinic Cu_2_V_2_O_7_ and Co(ii) doped Cu_2_V_2_O_7_ (Co_5%_–CuV, Co_10%_–CuV, and Co_20%_–CuV) have been developed through a facile solid-state synthesis strategy and comprehensively demonstrated as an efficient electrocatalyst for the enhanced HER and glycerol oxidation performance. Among the synthesized samples, Co_10%_–CuV exhibits the best electrocatalytic HER and GOR activity, where substantially low overpotential requirements of 176 mV and 160 mV were observed, respectively, for achieving 10 mA cm^−2^ current density in 1.0 M KOH solution. The in-depth analysis of the products *via* NMR indicates the generation of formate as the preferred pathway during the oxidation of glycerol with this newly developed Co(ii)-doped copper vanadate electrode assembly. This robust, easily synthesizable, and dual-active material can be deployed on both sides of a two-electrode electrolyzer for facile oxidation of glycerol along with simultaneous production of H_2_ in alkaline water media. Hence, this Co_10%_–CuV can form the crux of low-cost catalytic materials driving a carbon neutral biomass to H_2_ conversion assembly aiding our pursuit of a sustainable energy paradigm.

## Author contributions

V. T., L. S. P. and S. J. conceptualized the project; V. T., S. J., and A. D. performed the experiments; V. T., S. J. D. K. and A. D. analyzed the data; S. J., A. D. and V. T. wrote the manuscript; V. T., S. J. and A. D. revised and edited the manuscript; S. J. and A. D. supervised the project.

## Conflicts of interest

The authors declare no competing financial interest.

## Supplementary Material

NA-005-D2NA00724J-s001
