# Supplementary material for: Cobalt-doped copper vanadate: a dual active electrocatalyst propelling efficient H2 evolution and glycerol oxidation in alkaline water
Source: Nanoscale Adv. 2022 Nov 25;5(1):237–46. doi: 10.1039/d2na00724j (PMC9765594; doi:10.1039/d2na00724j)
Supplement: NA-005-D2NA00724J-s001 [file NA-005-D2NA00724J-s001.pdf]

Electronic Supplementary Material (ESI) for Nanoscale Advances.
This journal is © The Royal Society of Chemistry 2022

Supporting Information

for

Cobalt-doped copper vanadate: A dual active electrocatalyst propelling efficient H_2 evolution and glycerol oxidation in alkaline water

Vijay Tripathi[a§], Siddarth Jain[a§*], Dinesh Kabra[b], Leela S. Panchakarla[a], Arnab Dutta[a,c,d*]

[a] *Department of Chemistry, Indian Institute of Technology, Bombay, 400076, India*

[b] *Department of Physics, Indian Institute of Technology, Bombay, 400076, India*

[c] *Interdisciplinary Program in Climate Studies, Indian Institute of Technology, Bombay, 400076, India*

[d] *National Center of Excellence in CCU, Indian Institute of Technology, Bombay, 400076, India*

[§]These two authors have contributed equally

*Corresponding author: jainsiddarth477@gmail.com; arnab.dutta@iitb.ac.in

Table of Contents

Details	Page No
Materials and Experimental Details	S3-S5
XRD spectra of Pure Copper vanadate and doped copper vanadate	S6
SEM Images of pure CuV and Co (II) doped CuV	S7
EDX spectrum of $Co_{10\%}$-CuV	S7
Full range XPS survey spectrum of $Co_{10\%}$-CuV	S8
Comparative LSV curves showing HER activity of $Co_{10\%}$-CuV before and after 1 hr of electrolysis	S8
Cyclic voltammograms at various scan rate in the non-Faradaic region	S9
Tauc-plot of Co^{2+} doped copper vanadates	S10
LSV curve showing comparison of glycerol oxidation at variable Co^{2+} doped CuV	S11
EIS spectrum of $Co_{10\%}$-CuV modified electrode	S11
ECSA Normalized LSV polarization curve	S12
Comparative Turn over frequency (TOF)	S12
^{1}H-NMR graph of formic acid with various concentration	S13
^{13}C-NMR graph after bulk electrolysis of glycerol	S13
Long term I-t analysis of $Co_{10\%}$-CuV modified electrode	S14
LSV curve of $Co_{10\%}$-CuV modified electrode obtained after long term I-t analysis	S14
Recyclability of $Co_{10\%}$-CuV modified electrode	S15
Comparison table showing HER activity of various catalyst.	S16
Comparison table showing GOR activity of various catalyst	S16
References	S17

Materials and Experimental Details

Materials: Copper nitrate ($Cu(NO_3)_2$. $4H_2O$) (95%, Loba Chemie Pvt. Ltd.) Vanadyl sulphate ($VOSO_4$) (97%, Sisco Research Laboratories Pvt. Ltd), Cobalt nitrate ($Co(NO_3)_2$.$6H_2O$)(95%, Loba Chemie Pvt. Ltd.), KOH (85%, Loba Chemie Pvt. Ltd.), Glycerol($C_3O_3H_8$) (99%, Sigma-Aldrich.)

Analytical tools: X-ray diffraction (Rigaku, Cu−Kα radiation 1.514 Å), Scanning electron microscopy (SEM, JEOL JSM-7600F FEG-SEM), X-ray photoelectron spectroscopy (Axis Supra Model, SHIMADZU group), Transmission electron microscope (Thermo Scientific, Themis 300 G3), Electrochemical work station (Autolab), Gas-chromatography (Dhruva CIC), Nuclear magnetic resonance (Bruker Avance III 400), ICP-AES (Arcos)

Synthesis of copper vanadate and Co doped copper vanadate

Synthesis of copper vanadate (CuV)

Under inert conditions, copper vanadate was synthesised employing the high-temperature solid-state method. In a 5:2 molar ratio, $Cu(NO_3)_2$ and $VOSO_4$ were dissolved in milli-Q water and stirred for 2 hours to form a homogenous solution. The dried sample was ground in agate mortar and pestle to obtain fine powder. The fine powder of solid precursor was then annealed for 2 hours in the tube furnace at 550 °C in an argon environment.

Synthesis of cobalt doped copper vanadate ($Co_{x\%}$-CuV)

In order to synthesize variable ratios of cobalt doped copper vanadate, molar equivalents of $Co(NO_3)_2$ nitrate in varying ratios of 5, 10, and 20% were dissolved and agitated in milli-Q water for 2 hours with $Cu(NO_3)_2$ and $VOSO_4$. The subsequent steps followed were same as for the synthesis of copper vanadate.

Electrochemical studies: All electrochemical experiments were performed on autolab electrochemical analyzer. A three-electrode system had been organized using glassy carbon as working electrode, Hg/Hg_2Cl_2 (standard calomel electrode) as reference electrode and Pt as a counter electrode. 1. 0 M KOH solution used as electrolyte for all the measurement. 5.0 mg /mL of copper vanadate and Co^{2+} doped copper vanadate was dispersed in the IPA with nafion binder and sonicated it for 10 mins. The dispersed catalyst (10 μL) was drop casted on the glassy carbon electrode and dried under IR lamp for 30 mins. All the potentials reported in this work here converted vs RHE in 1.0 M KOH.

E *vs.* RHE = E *vs.* Ag/AgCl + 0.197 + 0.0591 × pH

The linear sweep voltammetry (LSV) were measured at the scan rate of 5 mVs^{-1}.

Product Analysis:

Formic Acid:

To identify the by-products of glycerol oxidation, a prolonged electrolysis experiment was conducted. After electrolysis, the obtained electrolytes were analysed using a nuclear magnetic resonance (NMR) spectrometer. All liquids to be examined included 540 ml of electrolyte and 60 ml of D_2O, and their ^{1}H and ^{13}C NMR were determined. Formic acid (20–50 mM for 1H NMR) was dissolved in 1.0 M KOH aqueous solution to form a formic acid standard, and the ^{1}H NMR spectra of the standard were obtained under the same circumstances as solution prepared by electrolyzing biomass with glycerol. Gas chromatography was used to glance at the gaseous products that came from the cathode after electrolysis. Giving to the hypothesised chemical pathway, the transformation of glycerol to formic acid may be represented by the following equation:

$C_3H_8O_3$ (glycerol) - $8e^-$ + $8OH^-$ - $5H_2O$ = $3CH_2O_2$

Consequently, it is possible to calculate formic acid's faradaic efficiency for glycerol oxidation by following equation:

$$FE(\%) = \frac{N\ (Formic\ acid\ yeild)}{Q\ total/(Z \times\ F)} \times 100$$

Where, Q total is the total charge passed through the electrodes, Z= 8/3 is the number of electrons that form a mole of formic acid, and F is the Faraday constant (96485 C mol^{-1}).

H_2 production calculation:

The Faraday efficiency (%) of the H_2 production can be determined by the following Equation:

$$FE(\%) = \frac{N\ (Hydrogen\ production)}{Q\ total/(Z \times\ F)} \times 100$$

Here, Q = Total charge passed, z = 2 is the number of electrons that produce a molecule of H_2, and F is the Faraday constant (96485 C mol^{-1}).

Electrochemical Active Surface Area (ECSA):

ECSA was calculated by measuring double-layer capacitance (C_{dl}) from cyclic voltammograms (CVs) recorded within the non-Faradaic potential window at different scan rates (10 to 70 mV s^{-1}). The C_{dl} value was calculated as the slope of a straight line based on the plot of current density vs scan rate. Using the specific capacitance of 0.04 mF cm^{-2} for an ideal flat surface, it is possible to derive the electrochemical active surface area.

$$A_{\mathrm{ECSA}} = \frac{\mathrm{C_{dl}}\,(\mathrm{catalyst})}{0.04\ \mathrm{mF\,cm^{-2}}}$$

Turnover Frequency: TOF of the reaction was calculated by the following equation

$$\mathrm{TOF} = \frac{j \times A_{\mathrm{geo}}}{n \times F \times N_{\mathrm{site}}}$$

where j is the current density (A/cm^2) at @1.6 V vs RHE, A_{geo} is the geometric area of the electrode, n is the number of electrons involved, F is the Faraday constant, and N_{site} is the total number of metal sites (mole) on the electrode determined by ICP-AES.[13]

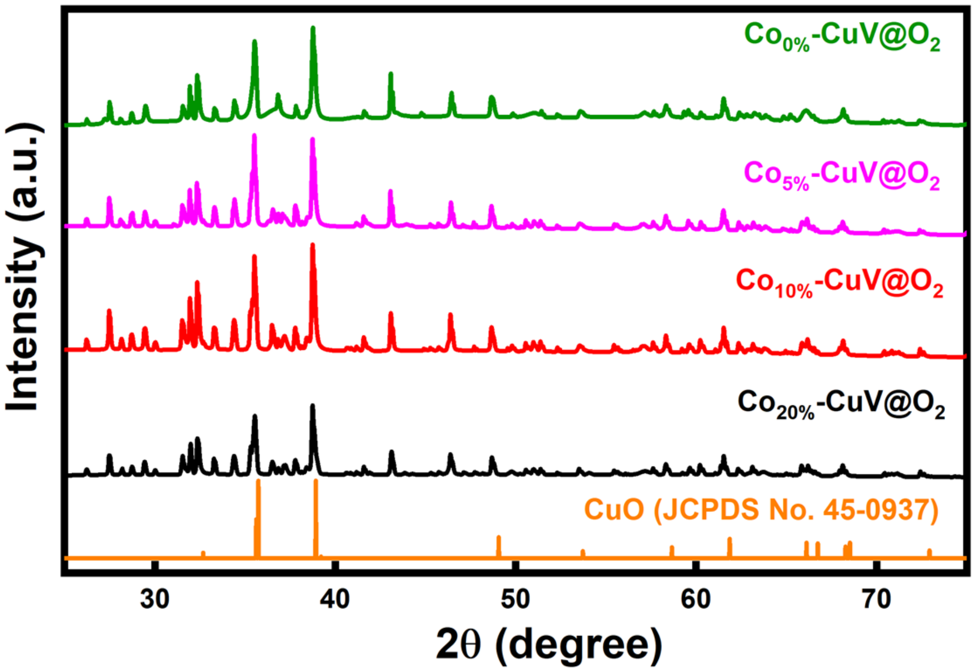

Figure S1. The comparative XRD spectra of pure Copper vanadate and doped copper vanadate obtained at 550 °C under oxygen atmosphere.

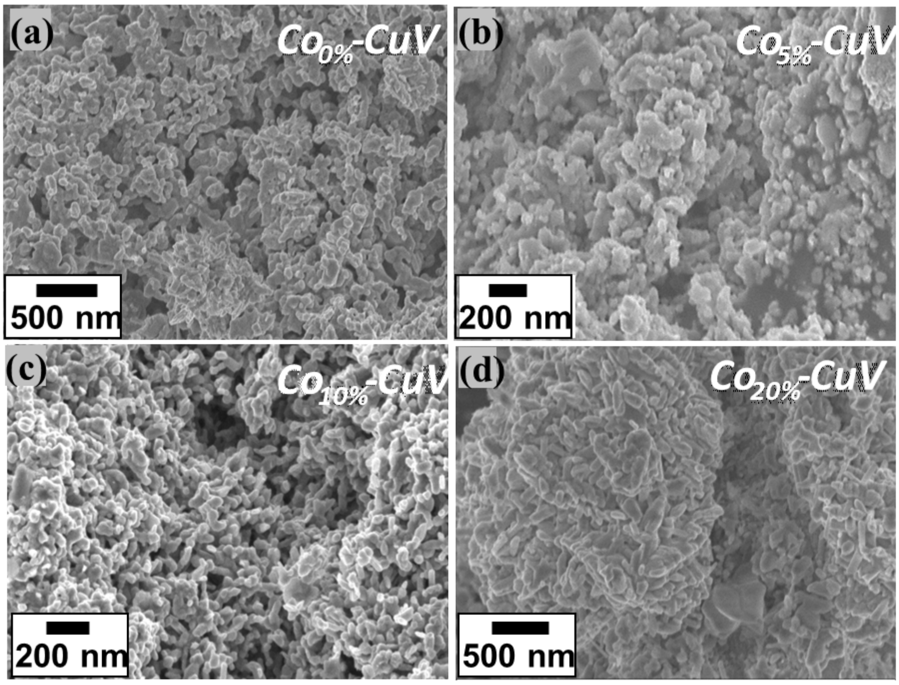

Figure S2. SEM images of (b) $Co_{0\%}$-CuV, (c) $Co_{5\%}$-CuV, (d) $Co_{10\%}$-CuV and (e) $Co_{20\%}$-CuV obtained at 550°C under inert atmosphere.

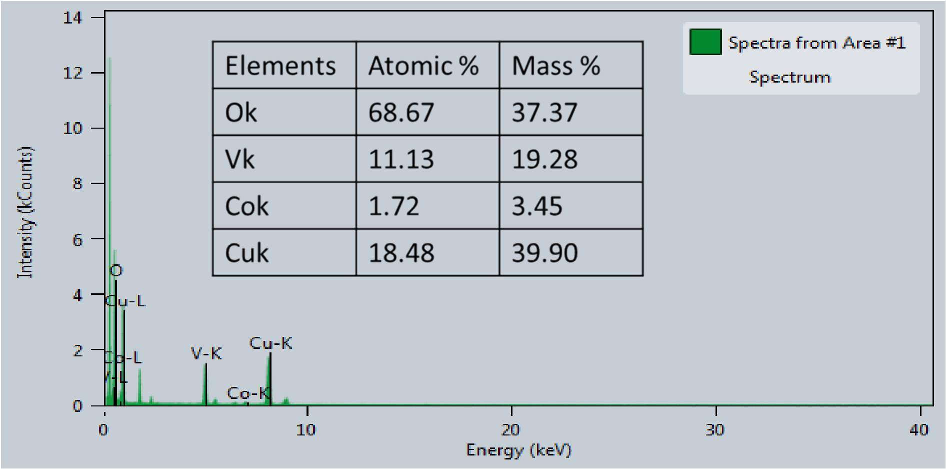

Elements	Atomic %	Mass %
Ok	68.67	37.37
Vk	11.13	19.28
Cok	1.72	3.45
Cuk	18.48	39.90

Figure S3. EDS spectrum of $Co_{10\%}$-CuV obtained at 550°C under inert atmosphere.

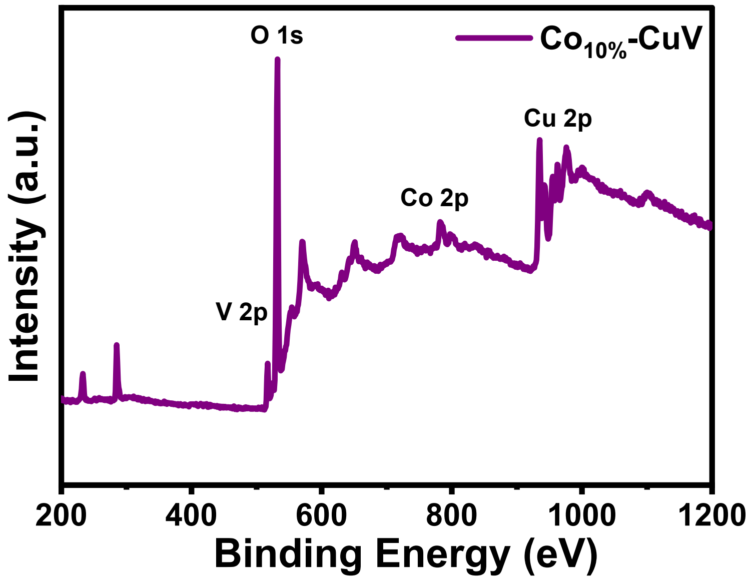

Figure S4. The full range XPS survey spectrum of the 10% Co (II)-doped $Cu_2V_2O_7$ ($Co_{10\%}$-CuV) sample prepared at 550 °C under inert atmosphere.

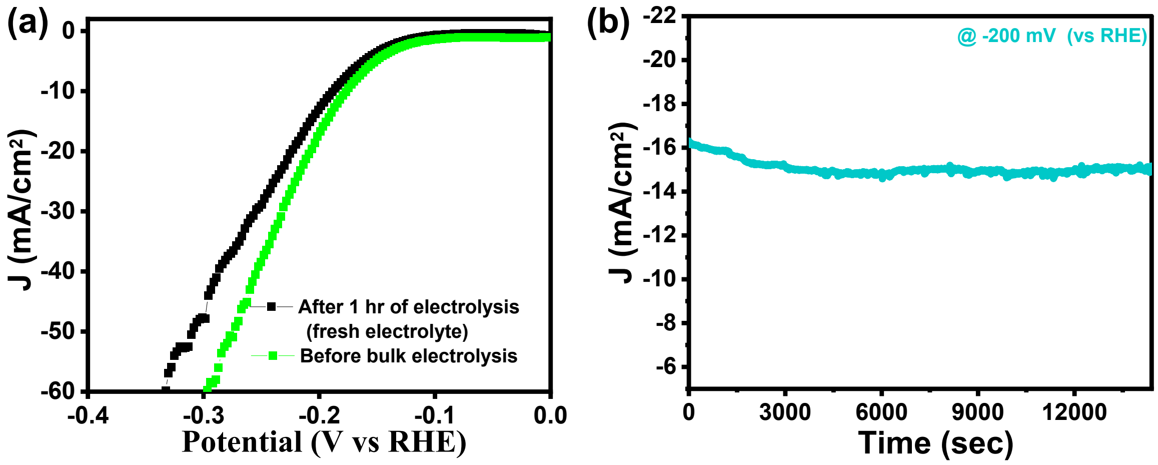

Figure S5. (a) Comparative LSV curves showing HER activity of $Co_{10\%}$-CuV before and after 1 hr of electrolysis in 1.0 M KOH. Scan rate 5 mV/s (b) Long term chronocoulometric experiment performed with $Co_{10\%}$-CuV/GCE modified electrode showing stability of electrode in 1.0 M KOH, with an applied potential of -0.2 V vs RHE, at room temperature.

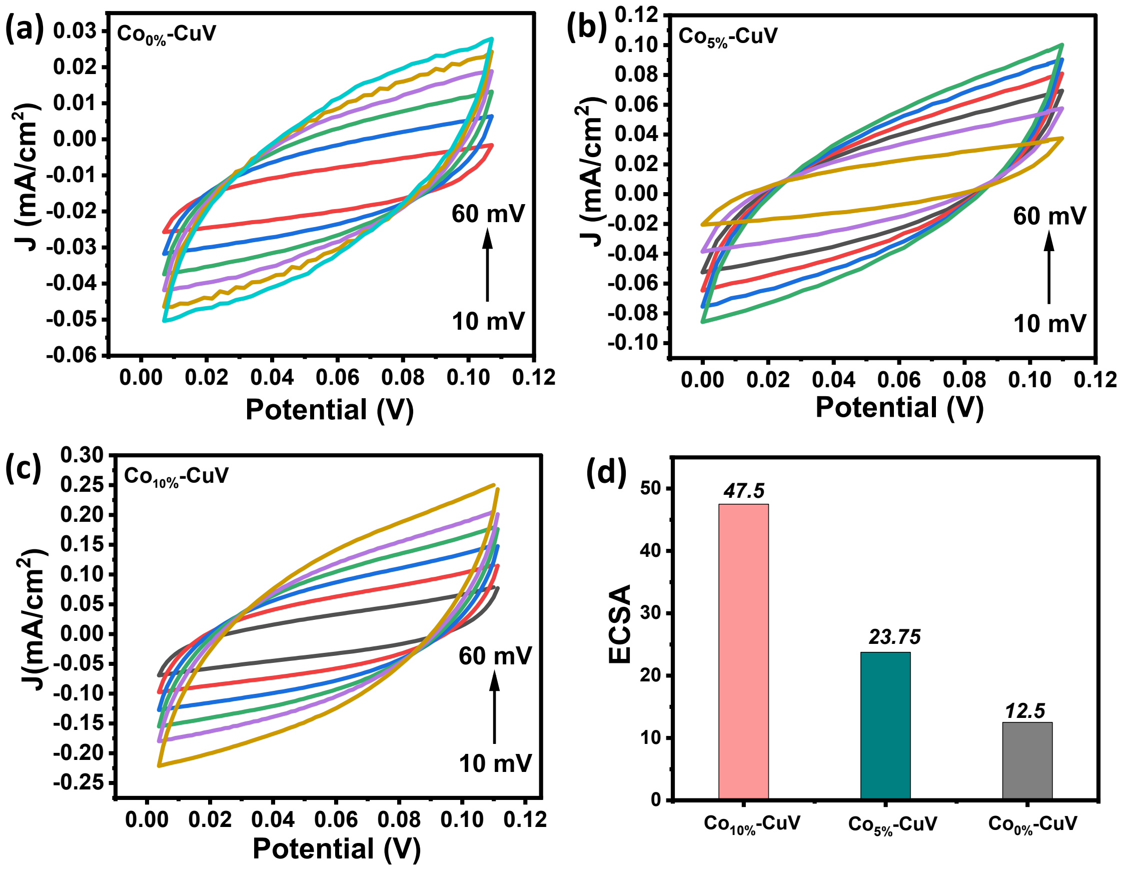

Figure S6: (a-c) Cyclic voltammograms at various scan rate in the non-Faradaic region of 0 to 0.1V vs. Ag/AgCl (d) Bar plot showing ECSA value of Co(II) doped and undoped copper vanadate

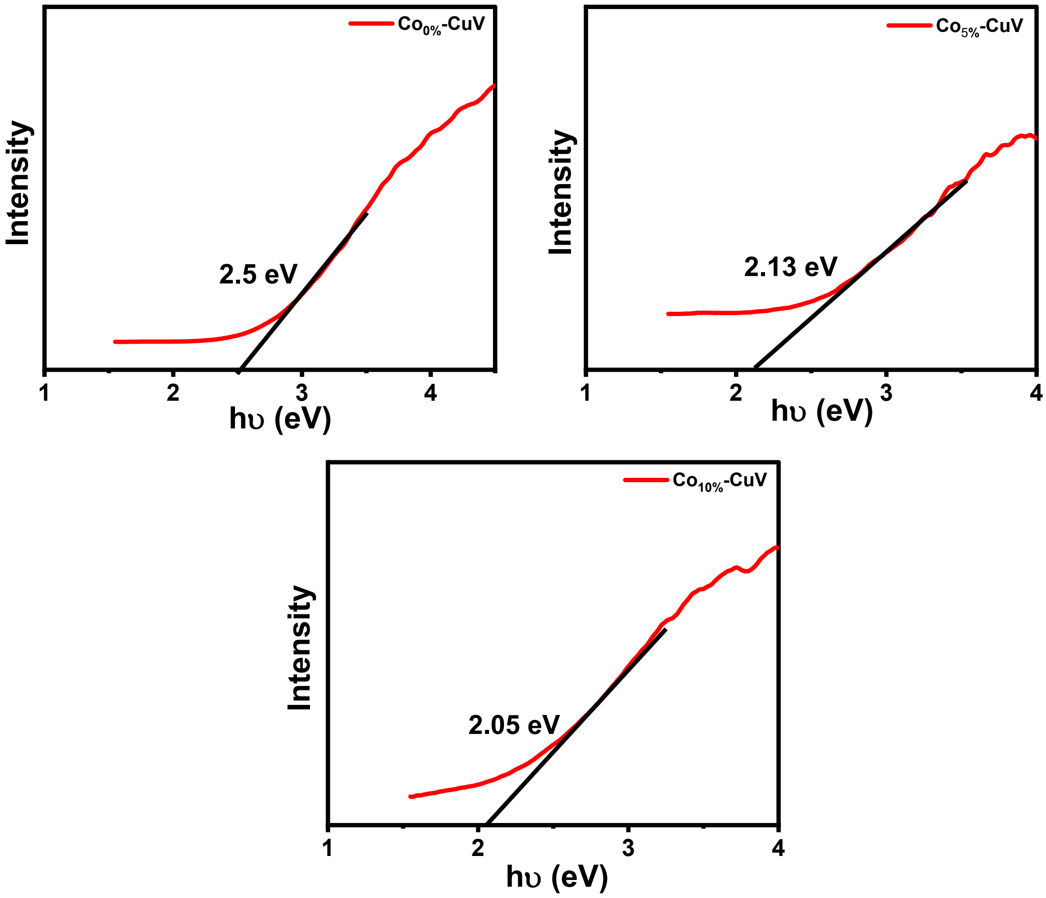

Figure S7. The Tauc-plots of various Co-doped copper vanadates showcasing their respective optical band gaps.

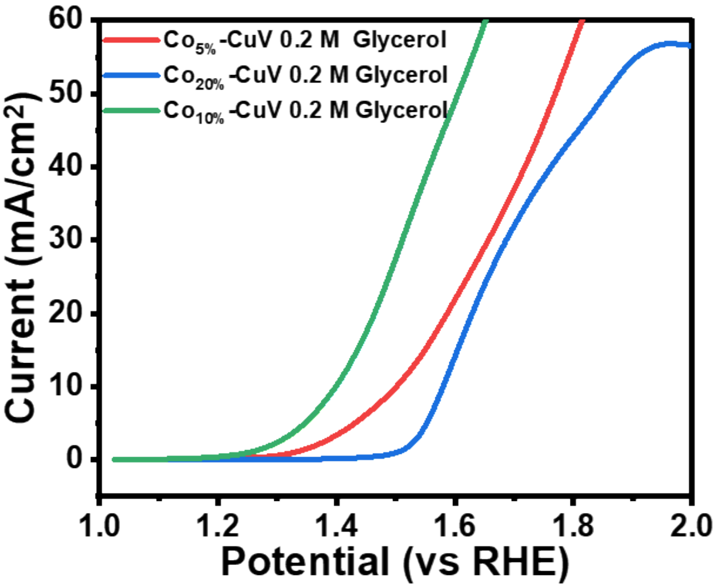

Figure S8. Comparative LSV curves showing glycerol oxidation signatures for $Co_{5\%}$-CuV (red trace), $Co_{10\%}$-CuV (green trace), and $Co_{20\%}$-CuV (blue trace) in the presence of 0.2 M glycerol in 1.0 M KOH. Scan rate 5 mV/s. Data recorded at 298 K under anaerobic conditions.

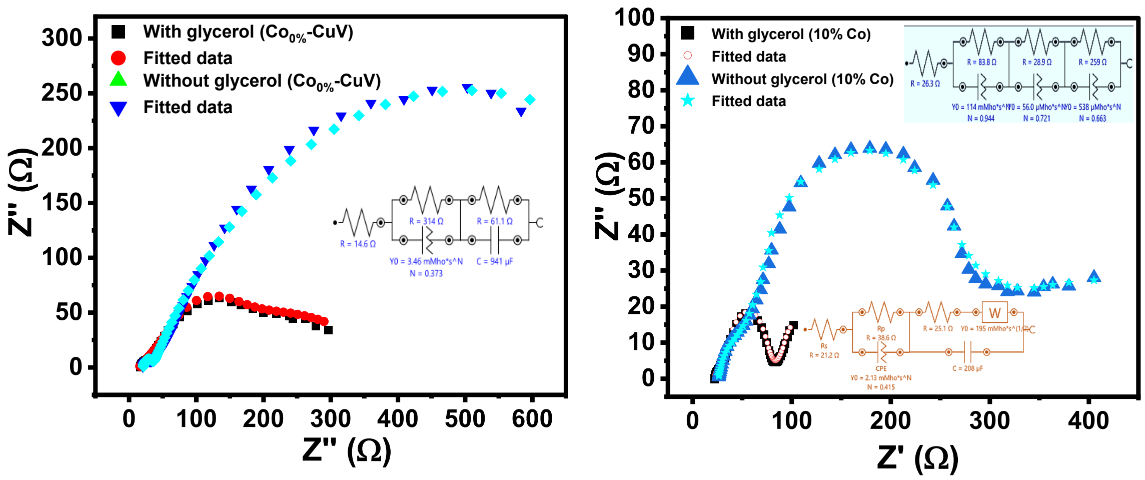

Figure S9. The comparative EIS spectra of $Co_{0\%}$-CuV and $Co_{10\%}$-CuV modified electrode in the presence (red trace) and absence of glycerol (blue trace), under an open circuit potential with an input frequency from 100 kHz to 0.01 Hz, in 1.0 M KOH solution.

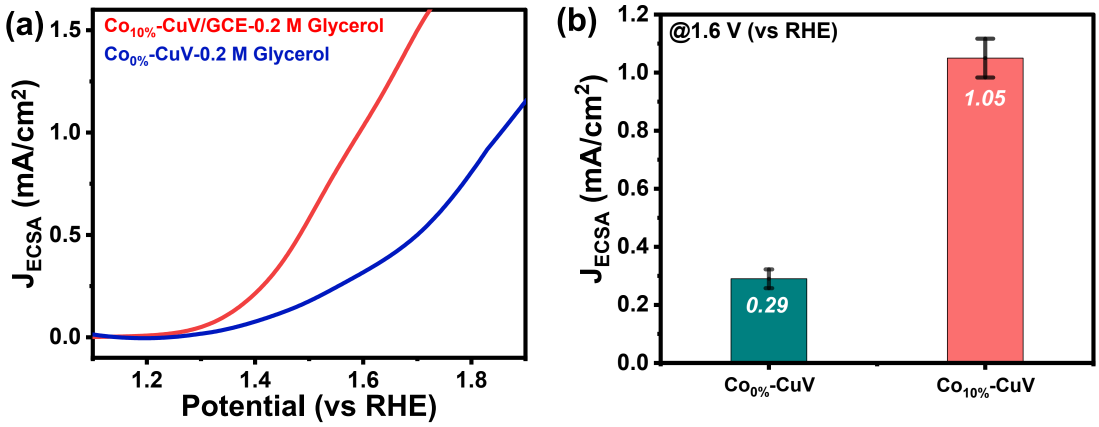

Figure: S10 : (a) ECSA Normalized LSV polarization curve of $Co_{0\%}$-CuV and $Co_{10\%}$-CuV modified electrode (b) Comparative ECSA normalized current densities measured at 1.6 V for $Co_{0\%}$-CuV (Green-striped column), and $Co_{10\%}$-CuV (Red-striped column). Standard error was determined by taking measurements three times.

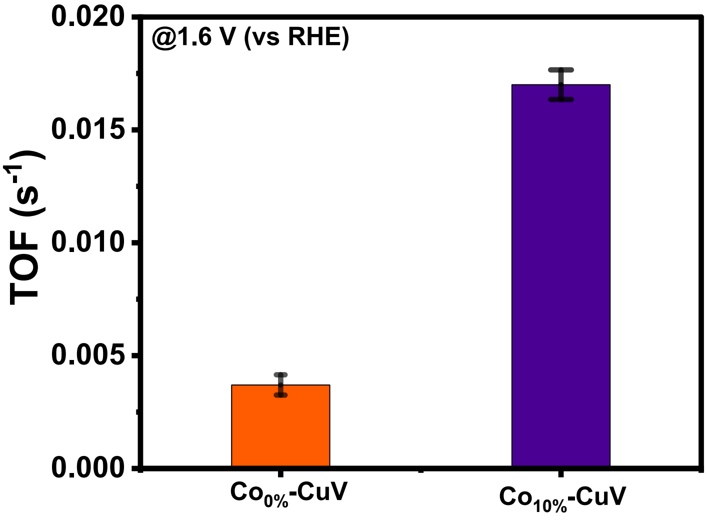

Figure: S11 Comparative Turn over frequency (TOF) measured at 1.6 V for $Co_{0\%}$-CuV (Orange-striped column), and $Co_{10\%}$-CuV (Purple-striped column). Standard error was determined by taking measurements three times.

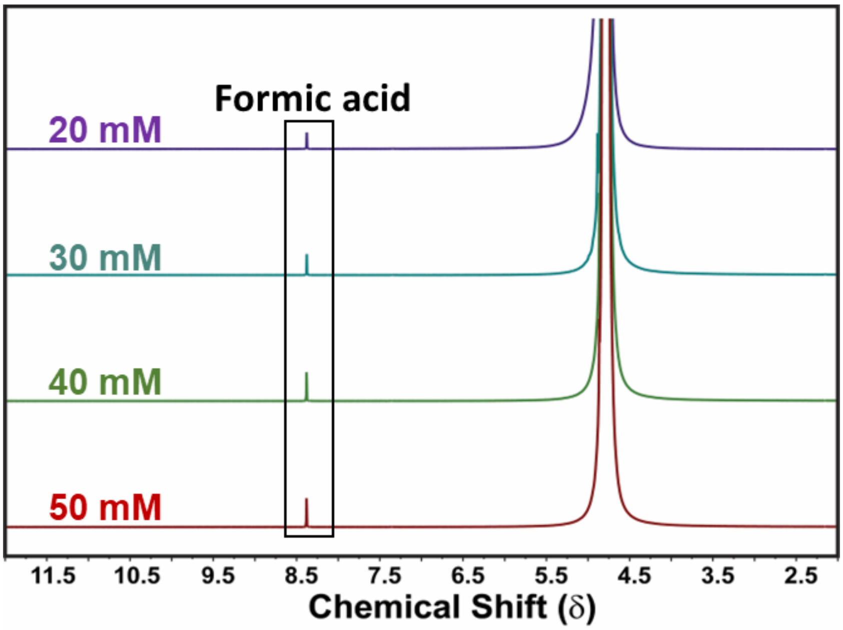

Figure S12. ^{1}H-NMR data recorded at variable concentration of formic acid in D_2O solvent at 298 K.

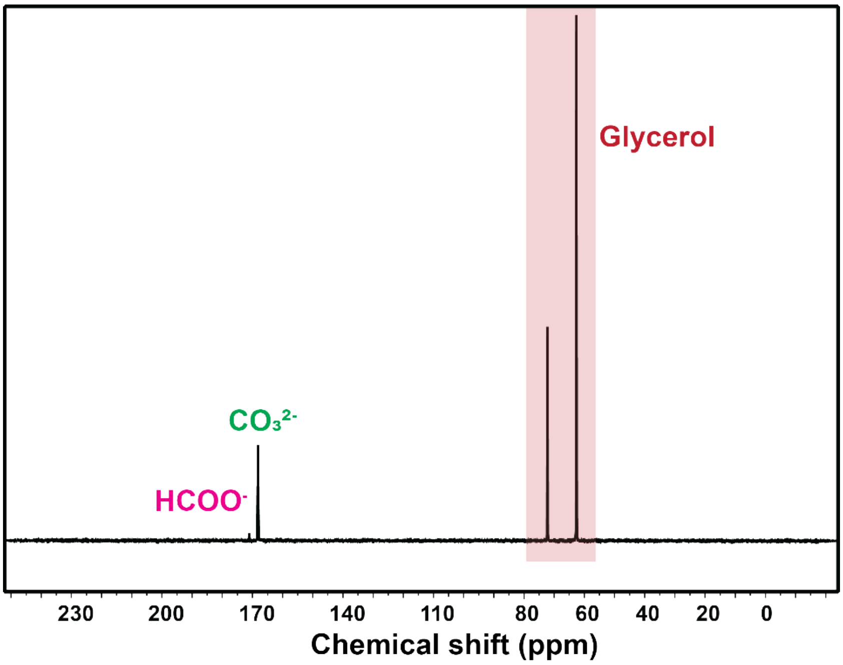

Figure S13. ^{13}C-NMR data recorded after 12 hours of electrolysis of glycerol in D_2O solvent at 298 K.

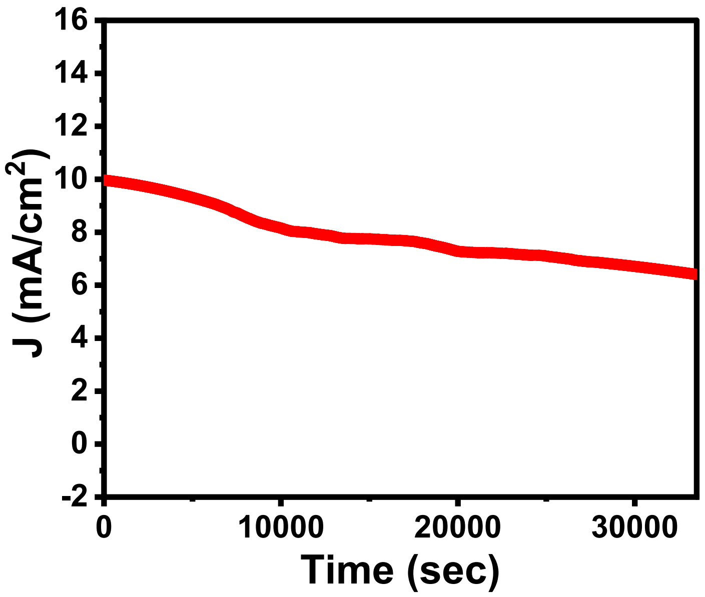

Figure S14. Long term chronocoulometric experiment performed with $Co_{10\%}$-CuV/GCE modified electrode in the presence of 0.2 M glycerol showing stability of electrode in 1.0 M KOH, with an applied potential of 1.5 V vs RHE, under inert condition at room temperature.

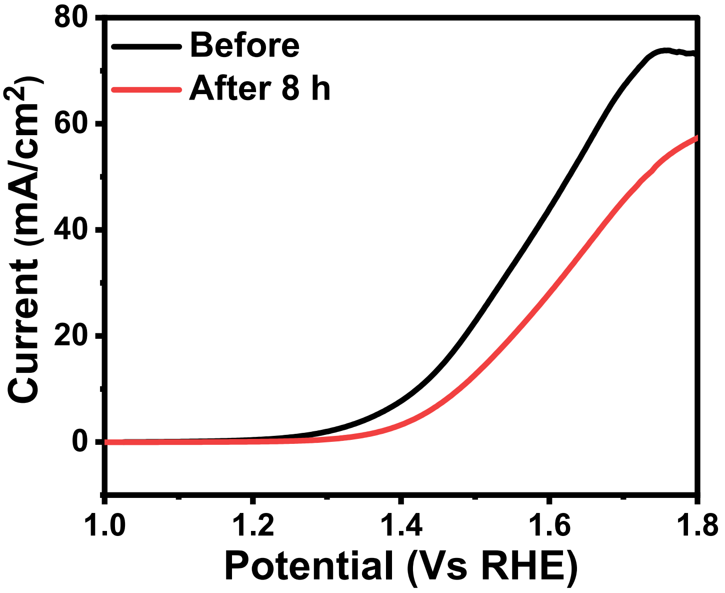

Figure S15. LSV curve of $Co_{10\%}$-CuV/GCE modified electrode obtained after 8 hours of bulk electrolysis in the presence of 0.2 M glycerol in 1.0 M KOH at room temperature.

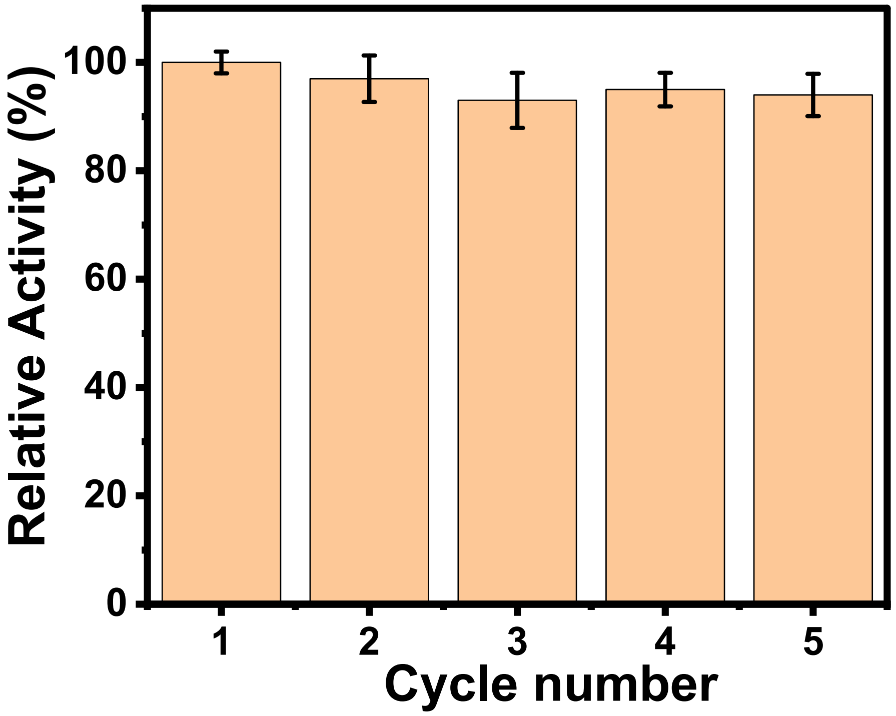

Figure S16. Recyclability of $Co_{10\%}$-CuV/GCE modified electrode (n=3) in the presence of 0.2 M glycerol in 1.0 M KOH solution.

Table S1: Comparison table showing HER activity of various catalysts.

Material	Electrode/Substrate	Electrolyte	η_{HER} (10 mA/cm^2)	Tafel slope (mV dec^{-1})	Ref.
Cu_2S micro hexagons	Graphite rod	0.5 M H_2SO_4<br>1 M KOH	312<br>330	49.79<br>106.93	1
Cu_2MOS_4	Glassy carbon	0.5 M H_2SO_4	300	64.3	2
Cu_3P@C	Carbon fiber paper	0.5 M H_2SO_4	203	83	3
Cu-Co-P@$Cu_2CO_2O_4$	Ti mess	1.0 M KOH	104	74	4
Co@Co_3O_4–Nano carbon	Nickle Foam	1.0 M KOH	221	77.3	5
Co_3O_4/Ppy/MWCNT	Glassy carbon	1.0 M KOH	490	110	6
CCO-1.5−5 h-etch	Glassy carbon	1.0 M KOH	320	68	7
**$Co_{10\%}$-CuV**	**Glassy carbon**	**1.0 M KOH**	**176**	**94**	**This work**

Table S2: Comparison table showing GOR or COR (chemical oxidation reaction) activity of various catalysts.

Catalyst	Electrolyte	Major product	Onset V OER	Onset V COR/GOR	Ref
N-CoOx anode Si NW-Pt photocathode	1 M KOH+1 M glycerol	Formic acid	1.58	1.31	8
Ni-Mo-N/CFC anode Ni-Mo-N/CFC cathode	1 M KOH+0. 1 M glycerol	Formic acid	1.57	1.30	9
$CuCo_2O_4$ anode	0.1 M KOH+0. 1 M glycero	Formic acid	1.55	1.26	10
Ni_3S_2/NF	1 M KOH+10 mM HMF	FDCA/H_2	1.58	1.46	11
Ni_2P/Ni/NF anode	1M KOH +30mM Furfural	Furoic acid	1.55	1.43	12
$Co_{10\%}$-CuV	1 M KOH+0.2 M glycerol	Formic acid	1.61	1.39	This work

References:

1. K. Bhat, N. Hosakoppa, *J. Sci. Adv. Mater. Devices* 2020, ***5***, 361–367.
2. D. P. Tran, M. Nguyen, S. S. Pramana, A. Bhattacharjee, S. Y. Chiam, J. Fize, M. J. Field, V. Artero, H. L. Wong, J. Loo, J. Barber, *Energy Environ. Sci.* 2012, ***5***, 8912–8916.
3. M. Pi, T. Yang, S. Wang, S. Chen, *J. Mater. Res.* 2018, ***33***, 548–556.
4. Zheng, X.; Zhang, J.; Sun, Z.; Zhang, Z.; Xi, D. *Eur. J. Inorg. Chem.* 2018, 3565–3569
5. C. Bai, S. Wei, D. Deng, X. Lin, M. Zheng, Q. Dong, *J. Mater. Chem. A* 2017, *5* (20), 9533−9536.
6. S. S. Jayaseelan, N. Bhuvanendran, Q. Xu, H. Su, *Int. J. Hydrogen Energy* 2020, ***45*** (7), 4587−4595.
7. K. S. Roy, C. Subramaniam, L. S. Panchakarla, *ACS Appl. Mater. Interfaces* 2021, ***13*** (8), 9897–9907.
8. Z. Ke, N. Williams, X. Yan, S. Younan, D. He, X. Song, X. Pan, X. Xiao, J. Gu, *J. Mater. Chem. A* 2021, ***9*** (35), 19975–19983.
9. J. Zhang, Y. Shen, *J. Electrochem. Soc.* 2021, ***168*** (8), 084510.
10. X. Han, H. Sheng, C. Yu, T. W. Walker, G. W. Huber, J. Qiu, S. Jin, *ACS Catal.* 2020, ***10*** (12), 6741–6752.
11. N. Jiang, X. Liu, J. Dong, B. You, X. Liu, Y. Sun, *ChemNanoMat* 2017, ***3*** (7), 491–495.
12. B. You, X. Liu, N. Jiang, Y. Sun, *J. Am. Chem. Soc.* 2016, ***138*** (41), 13639–13646.
13. S. K. T. Aziz, M. Ummekar, I. Karajagi, S. K. Riyajuddin, K. V. R. Siddhartha, A. Saini, A. Potbhare, R. G. Chaudhary, V. Vishal, P. C. Ghosh and A. Dutta, *Cell Rep. Phys. Sci.*, 2022, **3**, 101106.
